# Recovery of whole mitochondrial genome from compromised samples via multiplex PCR and massively parallel sequencing

**DOI:** 10.4155/fsoa-2018-0059

**Published:** 2018-08-24

**Authors:** Maureen P Hickman, Kelly S Grisedale, Brittania J Bintz, Erin S Burnside, Erin K Hanson, Jack Ballantyne, Mark R Wilson

**Affiliations:** 1 Forensic Science Program, Western Carolina University, Cullowhee, NC 28723, USA; 2 National Center for Forensic Science, University of Central Florida, Orlando, FL 23826, USA; 3 Department of Chemistry, University of Central Florida, Orlando, FL 23816, USA; 4 MRW Analytics, Fredericksburg, VA 22405, USA

**Keywords:** degraded DNA, massively parallel sequencing, mitochondrial genome

## Abstract

In forensic casework, compromised samples often possess limited or degraded nuclear DNA, rendering mitochondrial DNA a more feasible option for forensic DNA analyses. The emergence of massively parallel sequencing (MPS) has enabled the recovery of extensive sequence information from very low quantities of DNA. We have developed a multiplex PCR method that amplifies the complete mitochondrial genome in a range of forensically relevant samples including single cells, cremated remains, bone, maggot and hairs isolated from dust bunnies. Following library preparation, MPS yields complete or nearly complete mitochondrial genome coverage for all samples. To confirm concordance between sample types and between sequencing platforms, we compared sequencing results from hair and buccal swabs from two references. Low initial DNA input into the multiplex PCR allows for conservation of precious DNA while MPS maximizes recovery of genetic information.

## Lay abstract

Compromised samples are routine in forensic casework, and evidence DNA is often limited and/or degraded. Mitochondrial DNA is often a suitable option for forensic analysis as it is available in multiple copies per cell. We have established a method that amplifies the complete mitochondrial genome in a range of compromised samples and utilizes new high-throughput sequencing technologies to recover maximal genetic information from a small amount of sample. Combination of the aforementioned amplification method and high-throughput sequencing allows the analyst to conserve DNA in forensic cases where DNA is often limited.

The enhancement of mitochondrial DNA (mtDNA) typing over the last 25 years has distinguished it as a viable application in forensic casework, particularly when dealing with compromised samples, which often contain degraded nuclear DNA [1–3]. mtDNA is available in multiple copies per cell and confers an increased detection sensitivity compared with nuclear DNA [4,5]. Traditionally, studies have focused on the noncoding control region (CR) of the mitochondrial genome (mtGenome), which spans over 1100 base pairs and includes two hypervariable regions. There are cases, however, when the CR does not provide adequate discriminatory power for identification [6]. In cases where identity cannot be resolved through CR sequencing, analysis of the complete mtGenome may provide greater power of discrimination [7].

Currently Sanger-type sequencing (STS) is the method used in most casework laboratories. Although STS has been traditionally considered the gold standard, it is labor intensive, expensive and requires significant initial DNA input, which is not ideal when forensic sample DNA is frequently limited. Massively parallel sequencing (MPS) technologies offer an efficient way to generate high-throughput, in-depth, sequence information and have recently proven successful in recovering significant genomic DNA sequences from compromised sample types [8–10].

Nearly 25 years after validation of mtDNA sequencing for forensic casework, laboratory methods for mtDNA analysis have changed very little. Here we present a novel multiplex PCR method for amplifying the full mtGenome from a variety of forensically relevant samples, including hairs from dust bunnies, bones, cremated remains, maggots and single whole cells. Subsequent MPS yields complete or near complete mtGenome coverage of at least 100×.

Dust bunny hairs were collected from various household locations between 2008 and 2015 (Table 1). A human femur of unknown age was obtained from a commercial vendor (Skulls Unlimited, OK, USA) where the bone was cleaned of tissue and treated with a peroxide solution. Cremated remains, including highly calcined bone fragments, were obtained from a private donor. A maggot was collected from a donor at the Forensic Osteology Research Station (FOREST) at Western Carolina University, l NC, USA. DNA from hair shafts was extracted following a solid-phase protocol developed in-house [11]. DNA from remaining samples was extracted following protocols specialized for each sample type and are described in the associated protocol. Single whole cells were collected by micromanipulation [12] and amplified directly. Following DNA extraction, the mtGenome copy number was quantified using a quantitative PCR assay targeting a 105 base pair segment of the mtGenome [13]. For the multiplex PCR assay, we combined 46 previously published primer pairs (mitoSEQr Resequencing System, Applied Biosystems, CA, USA) into three 10 μl reactions (Figure 1). PCR conditions and thermal cycling parameters are provided in the associated protocol. Primer concentrations are provided in Supplementary Table 1.

**Table 1.** **Summary of relevant information and data collected for each sample.**

**Sample ID**	**Additional source information**	**mtGenome copy input**	**Average coverage**	**Percentage genome ≥100× coverage**	**Mitochondrial haplogroup**
***Challenging samples***					

Hair from dust bunny					

DBH-1	Living room (2015)	11,351	6177	100	H7c4

DBH-2	Bedroom (2008)	8592	8251	100	H11a1

DBH-3	Bathroom (2008)	2172	5396	100	C1b2^†^

DBH-4	Living room (2008)	4083	10,803	87	H7c4^†^

DBH-5	Bedroom (2014)	5362	10,512	100	H1b1(T16362C)

Bone					

FEM	Mid-shaft femur	3512	3251	96	U2b2^†^

Cremated remains					

R-1.1	Bone fragment	294,298	5092	98.6	U5b1c2^†^

R-1.2	Bone fragment	197	16,887	97.9	U5b1c2^†^

R-1.A	Ashes	1.54E6	14,721	99.1	U5b1c2^†^

Maggot					

MAG	FOREst donor	771,500	2691	58.4	H2a2a2^†^

Single cell					

FC-1	Buccal – female	N/A	1481	84.2	J1c4^†^

MC-1	Buccal – male	N/A	455	60.4	L2a1a2^†^

***Reference samples***					

Hair					

DH-1.1	Donor 1 (root)	12,911	20,676	100	J1c3e2

DH-1.2	Donor 1 (mid-shaft)	3179	13,603	100	J1c3e2

DH-2.1	Donor 2 (root)	6087	10,488	97.7	J1b1a1a

DH-2.2	Donor 2 (mid-shaft)	2205	1293	96.3	J1b1a1a

Buccal swab					

DBu-1	Donor 1 (swab)	4.07E7	5630	100	J1c3e2

DBu-2	Donor 2 (swab)	5.06E6	5063	97.6	J1b1a1a

Source information includes naming conventions, date, location and method of collection where applicable. mtGenome copy input indicates number of mtGenome equivalents input into each multiplex PCR. MPS metrics include average coverage across the mtGenome and percentage of the mtGenome covered by at least 100 reads. Mitochondrial haplogroup is the haplogroup assigned by the program mthap.

^†^Indicates imperfect match from assigned haplogroup.

DBH: Hair shaft collected from dust bunny; DBu: Reference donor buccal swab; DH: Reference donor hair shaft; FC: Single female buccal cell; FEM: Femor; FOREst: Forensic Osteology Research Station; MAG: Maggot; MC: Single male buccal cell; MPS: Massively parallel sequencing; mtGenome: Mitochondrial genome; R: Cremated remains.

**Figure 1.**
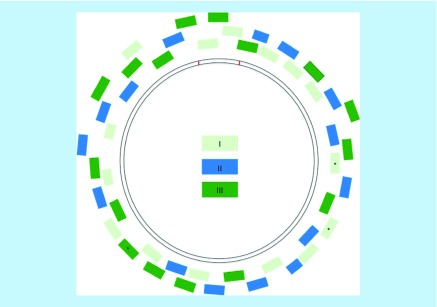
**Orientation of 46 primers around the mitochondrial genome.** Primers were multiplexed into three reactions (I, II, III). 43 primers were modified from Applied Biosystems MitoSEQr Kit (Thermo Fischer, MA, USA); starred (*) primers were separately redesigned.

We evaluated amplification success of each multiplex by identification of appropriate peak size and pattern on an Agilent 2100 Bioanalyzer (Agilent Technologies, CA, USA), and combined amplification products from each multiplex PCR were normalized to 0.2 ng/μl. All samples were subjected to library preparation following Nextera XT kit protocol (Illumina, CA, USA). MPS was performed on the Illumina MiSeq using paired-end 2 × 151 cycles. We performed secondary analysis using CLC Genomics Workbench version 8.5.1 (CLC bio, Aarhus, Denmark). Reads from each multiplex were simultaneously mapped to the revised Cambridge Reference Sequence (rCRS) and depth of coverage mapped across the mtGenome. We extracted a consensus sequence and uploaded FASTA files to a web-based mtDNA tool, mthap (https://dna.jameslick.com/mthap/), which compares sequences to the rCRS and evaluates sequence quality based on assignment to a mitochondrial haplogroup.

The number of mtGenome copies from challenging samples input into each multiplex PCR varied from 197 to 1.54 × 10^6^ copies (Table 1). The most variability in recovered mtDNA among sample types occurred among cremated remains, which originated from a single donor. High DNA recovery from cremated remains was not expected and could be due to variables such as short incineration time resulting in less degraded DNA or handling of remains by persons with improper protection resulting in contamination. We treated the outside of each burned bone fragment with 5% bleach solution and did not find evidence of contamination in our MPS results. Additionally, all cremated samples matched a reference sequence from a maternal line relative.

Average coverage ranged from 455× in the single male cell to 16887× in cremated remains (Table 1); however, samples were sequenced across multiple MiSeq runs with varying numbers of samples per run, which likely impacted coverage per sample. We attempted to evaluate coverage in a more informative way by assessing percentage of genome covered by a minimum number of reads. We set minimum coverage at 100× and found all but two samples (MAG and MC-1) had >80% genome coverage ≥100× (Table 1).

In order to assess concordance of MPS with traditional STS, we compared buccal and hair samples from two donors sequenced on both platforms. Sequences generated using MPS were concordant with STS with the exception of a 43/57% CT point heteroplasmy detected in MPS samples of Donor 1 but not detected in STS. Additionally, we compared sequences from mid-shaft hair samples to more robust root-end hair fragments and buccal samples. Sequence concordance was found in cases with coverage ≥100× for all samples. These results are consistent with a recently conducted study examining concordance and reproducibility of MPS of mtGenome using an Illumina MiSeq [14].

We used the mtDNA tool mthap to help provide an overview of sequence quality by evaluating sample fit into assigned mitochondrial haplogroups. Robust reference samples and some challenging samples were perfect or near perfect matches to their assigned haplogroups (Table 1). Some challenging samples were imperfect matches to their assigned haplogroups, containing missing or mismatched base calls. Such mismatched bases may be due to stochastic sampling effects exacerbated by PCR in low-copy number samples [15,16]. The patterns and magnitude of these incongruences warrant further investigation. The protocol for this method can be found in the Supplementary Material.

## Conclusion

We have established a method which amplifies the entire mtGenome in a range of challenging sample types ranging from dust bunny hairs to cremated remains. The method requires minimal DNA input, thereby conserving precious DNA and provides full mtGenome coverage of at least 100× in most samples. Multiplex PCR combined with MPS offers a promising new tool for maximizing genetic information.

## Future perspective

Massively parallel DNA sequencing is a powerful technique that has the potential to revolutionize forensic DNA typing. It offers the ability to generate expanded datasets in two complementary dimensions, one that easily extends the number of genetic targets amenable for typing, and a complementary depth dimension that provides replicate information at each sequenced base pair. Such data richness raises technical, philosophical, and interpretational issues that must be addressed before MPS is a routine technique in forensic science.

The wealth of data generated introduces a fundamental question as to how the data are to be treated. Some would argue that simplifying the data by using data divisors such as thresholds is the proper approach. The opposite perspective seeks to model the entire dataset. Variation within the data must also be addressed. For instance, sequencing reveals variants that are not observed using size-separation techniques, such as capillary electrophoresis. A question that may arise is whether or not these variants are a result of the amplification and sequencing process, and, if so, what are the implications of this for validly interpreting the MPS signal? With respect to human mitochondrial DNA analysis using MPS, the breadth dimension means that the entire mtDNA genome can be closed, and hence the power of this dimension is easily exploited. However, with this marker there are two sources of variation that must be further understood: biological variation present in the forensic sample that emanates from the sample donor and artifactual variation that results from the process of amplifying and sequencing the template molecules. These two sources must be more fully studied so that proper interpretational approaches can be developed. Once this is achieved, MPS can realize its huge promise in the field of forensic DNA typing.

**Executive summary**

- In forensic cases where DNA is limited or degraded, analysis of the mitochondrial genome can provide higher quality data than nuclear DNA.
- Analysis of the complete mitochondrial genome can provide greater power of discrimination than analysis of the control region alone.
- Massively parallel sequencing technologies have the ability to yield in-depth sequence information from compromised samples.
- We use a multiplex PCR method to amplify the complete mitochondrial genome in a variety of forensically relevant compromised samples and massively parallel sequencing to achieve complete or nearly complete coverage of the mitochondrial genome.
