# Supplementary material for: Recovery of whole mitochondrial genome from compromised samples via multiplex PCR and massively parallel sequencing
Source: Future Sci OA. 2018 Aug 24;4(9):FSO336. doi: 10.4155/fsoa-2018-0059 (PMC6222269; doi:10.4155/fsoa-2018-0059)
Supplement: Supplementary file 1 [file fsoa-04-336-s1.docx]

**Supplementary Table S1. Characterization of forty-six primer pairs**

| Multi-plex | WCU RSA | Primer Sequence 5’- 3’ | Size (bp) | Position in mtGenome (bp) | (nM) |
| --- | --- | --- | --- | --- | --- |
| I | 23 | F: GGTTGGTCAATTTCGTGCCAG  R: CTGCTAAATCCACCTTCGACCCTTAAG | 558 | 873 – 1431 | 200 |
|  | 1 | F: GCCCGTCACCCTCCTCAAGT  R: GGGATAGAGGGTCTGTGGGC | 593 | 1485 – 2078 | 300 |
|  | 3 | F: GCGTTCAAGCTCAACACCCA  R: GCAGGTTTGGTAGTTTAGGACCTGTG | 596 | 2201 – 2797 | 200 |
|  | 36* | F: CCCTCACCACTACAATCTTC  R: GGGCCCGATAGCTTATTTAG | 420 | 4013 - 4432 | 40 |
|  | 46* | F: CTCCACCTCAATCACACTAC  R: GTGAGGTAAAATGGCTGAGT | 533 | 5363 – 5895 | 300 |
|  | 27 | F: CAGCTCTAAGCCTCCTTATTCGAGC  R: CTGTTAGTAGTATAGTGATGCCAGCAGCTAGG | 542 | 5995 – 6537 | 300 |
|  | 39 | F: CAATTGGCTTCCTAGGGTTTATCGTG  R: GGGCATCCATATAGTCACTCCAGG | 660 | 6739 – 7399 | 200 |
|  | 29 | F: GAAAATCTGTTCGCTTCATTCATTGCC  R: GGTGGCGCTTCCAATTAGGTG | 527 | 8533 – 9060 | 100 |
|  | 31 | F: CGAGTCTCCCTTCACCATTTCCG  R: GGGTAAAAGGAGGGCAATTTCTAGATC | 528 | 9752 – 10280 | 200 |
|  | 8 | F: CTAGTCTTTGCCGCCTGCGA  R: GGGAAGGGAGCCTACTAGGGTGT | 577 | 10659 – 11236 | 300 |
|  | 33 | F: CAAACTACGAACGCACTCACAGTCG  R: GTCGTAAGCCTCTGTTGTCAGATTCAC | 440 | 11754 – 12194 | 80 |
|  | 34 | F: CCTTCTTGCTCATCAGTTGATGATACG  R: GCTTTGAAGAAGGCGTGGGTACAG | 558 | 12788 – 13346 | 200 |
|  | 13 | F: GCCATCGCTGTAGTATATCCAAAGACA  R: AGGCCTCGCCCGATGTGTAG | 598 | 14453 – 15051 | 200 |
|  | 44 | F: GAAAAAGTCTTTAACTCCACCATTAGCACC  R: GGGAACGTGTGGGCTATTTAGGCT | 587 | 15961 – 16548 | 200 |
|  | 22 | F: CAGGTCTATCACCCTATTAACCACTCACG  R: GGGTTGTATTGATGAGATTAGTAGTATGGGAG | 490 | 6 – 496 | 200 |
| II | 21 | F: CCCGTCCAGTGAGTCACCC  R: CCCAGTTTGGGTCTTAGCTATTGTGTG | 368 | 706 – 1074 | 200 |
|  | 19 | F: TGGCGGTGCTTCATATCCCTC  R: CGCCAGGTTTCAATTTCTATCGC | 596 | 1174 – 1770 | 200 |
|  | 4 | F: GCGGTACCCTAACCGTGCAA  R: GGGAAGGCGCTGTGAAGTAGG | 599 | 2571 – 3170 | 200 |
|  | 6 | F: CATACCCATGGCCAACCTCCT  R: CGGTTGGTCTCTGCTAGTGTGGA | 584 | 3306 – 3890 | 200 |
|  | 25 | F: CACCCCATCCTAAAGTAAGGTCAGC  R: GTTTGGTTTAATCCACCTCAACTGCC | 598 | 4389 – 4987 | 200 |
|  | 26 | F: CAGCTAAGCACCCTAATCAACTGGC  R: GGCCTCCACTATAGCAGATGCG | 567 | 5696 – 6263 | 200 |
|  | 38 | F: TGCCATAACCCAATACCAAACGC  R: CTTCCGTGGAGTGTGGCGAG | 467 | 6425 – 6892 | 40 |
|  | 45 | F: CCCGATGCATACACCACATGAA  R: CTAGGATGATGGCGGGCAGG | 572 | 7233 – 7805 | 200 |
|  | 28 | F: CTACGGTCAATGCTCTGAAATCTGTG  R: GTCATTGTTGGGTGGTGATTAGTCG | 510 | 8161 – 8671 | 200 |

|  | 17 | F: ATTGGAAGCGCCACCCTAGC  R: CAGGTGATTGATACTCCTGATGCGA | 597 | 9046 – 9643 | 200 |
| --- | --- | --- | --- | --- | --- |
|  | 32 | F: CTTATGACTCCCTAAAGCCCATGTCG  R: GTGATATTTGATCAGGAGAACGTGGTTAC | 536 | 11398 – 11934 | 200 |
|  | 10 | F: TTACCACCCTCGTTAACCCTAACAAA  R: CTGCTAGGAGGAGGCCTAGTAGTGG | 599 | 12395 – 12994 | 200 |
|  | 11 | F: GCAGCAGTCTGCGCCCTTAC  R: GCTGCCAGGCGTTTAATGGG | 514 | 13198 – 13712 | 200 |
|  | 12 | F: CAGCCCTCGCTGTCACTTTCC  R: GGATTGGTGCTGTGGGTGAAA | 571 | 13802 – 14373 | 300 |
|  | 15 | F: GACAGTCCCACCCTCACACGA  R: CGGATGCTACTTGTCCAATGATGG | 555 | 15257 – 15812 | 200 |
| III | 2 | F: AACTTTGCAAGGAGAGCCAAAGC  R: GCATGCCTGTGTTGGGTTGA | 568 | 1873 - 2441 | 200 |
|  | 5 | F: CCCTAGGGATACAGCGCATCCT  R: GCGGTGATGTAGAGGGTGATGG | 600 | 2927 – 3527 | 200 |
|  | 24 | F: CCTCTAGCCTAGCCGTTTACTCAATCC  R: GTGTATGAGTTGGTCGTAGCGGAATC | 538 | 3629 – 4167 | 80 |
|  | 37 | F: CTCTGAGTCCCAGAGGTACCCA  R: AGGTAGGAGTAGCGTGGTAAGGGC | 678 | 4805 – 5483 | 300 |
|  | 40 | F: GAGCTTATCACCTTTCATGATCACGC  R: GCTAAGTTAGCTTTACAGTGGGCTCTAG | 674 | 7640 – 8314 | 200 |
|  | 7 | F: CCTCCTCGGACTCCTGCCTC  R: TGAGGAGCGTTATGGAGTGGAAG | 561 | 8775 – 9336 | 60 |
|  | 30 | F: CGATACGGGATAATCCTATTTATTACCTCAG  R: TTATACTAAAAGAGTAAGACCCTCATCAATAG  ATGG | 561 | 9444 – 10005 | 350 |
|  | 9 | F: CCAACGCCACTTATCCAGCG  R: TGTCGTAGGCAGATGGAGCTTG | 596 | 10999 – 11595 | 200 |
|  | 41* | F: TTGACTACCACAACTCAACG  R: GGCCATATGTGTTGGAGATT | 605 | 10124 – 10728 | 200 |
|  | 18 | F: GGGCTCACTCACCCACCACAT  R: TGGGTTGTTTGGGTTGTGGCT | 553 | 12007 – 12560 | 80 |
|  | 42 | F: CCACATCATCGAAACCGCAAAC  R: GATGAGTGGGAAGAAGAAAGAGAGGAAG | 609 | 13515 – 14124 | 200 |
|  | 20 | F: ACGCCCATAATCATACAAAGCCC  R: GGGAGGTCGATGAATGAGTGGT | 587 | 14224 – 14811 | 200 |
|  | 14 | F: CGCCTGCCTGATCCTCCAA  R: GAAGGAAGAGAAGTAAGCCGAGGG | 595 | 14860 – 15455 | 200 |
|  | 16 | F: CTAGGAGGCGTCCTTGCCCT  R: GGGTTTGATGTGGGTTGGGTT | 577 | 15608 – 16185 | 200 |
|  | 43 | F: CCCCCCATGCTTACAAGCAAGT  R: CTGTGTGGAAAGCGGCTGTG | 635 | 16188 – 275 | 200 |
|  | 35 | F: TGGCCACAGCACTTAAACACATCTC  R: CTATTGACTTGGGTTAATCGTGTGACC | 606 | 321 – 927 | 200 |
| Primer sets are grouped in order of multiplex combination. RSA is the Resequencing Amplicon number corresponding to that in MitoSEQr™ resequencing system. Primer sequences, expected amplicon size, base pair position in human mtGenome, and concentration of each forward and reverse primer are given for each RSA.  *Indicates primer was redesigned. | | | | | |
